# Clinical significance of completion of radium-223 treatment and acute adverse events in patients with metastatic castration-resistant prostate cancer

**DOI:** 10.22038/AOJNMB.2022.67136.1468

**Published:** 2023

**Authors:** Kazuya Takeda, Yoshihide Kawasaki, Toru Sakayauchi, Chiaki Takahashi, Yu Katagiri, Takaya Tanabe, Yojiro Ishikawa, Keisuke Fujimoto, Masaki Kubozono, Maiko Kozumi, Keiko Abe, Kakutaro Narazaki, Shun Tasaka, Rei Umezawa, Takaya Yamamoto, Noriyoshi Takahashi, Yu Suzuki, Keita Kishida, So Omata, Akihiro Ito, Keiichi Jingu

**Affiliations:** 1 Department of Radiation Oncology, Tohoku University Graduate School of Medicine, Sendai, Japan; 2 Department of Radiation Oncology, South Miyagi Medical Center, Ogawara , Japan; 3 Department of Urology, Tohoku University Graduate School of Medicine, Sendai, Japan; 4 Department of Radiation Oncology, Osaki Citizen Hospital, Osaki, Japan; 5 Department of Radiation Oncology, Iwate Prefectural Isawa Hospital, Oshu, Japan; 6 Department of Radiation Oncology, Japanese Red Cross Ishinomaki Hospital, Ishinomaki, Japan; 7 Department of Radiation Oncology, Tohoku Rosai Hospital, Sendai, Japan; 8 Department of Radiation Oncology, Tohoku Medical and Pharmaceutical University Hospital, Sendai, Japan; 9 Department of Radiation Oncology, Iwaki City Medical Center, Iwaki, Japan; 10 Department of Radiation Oncology, Miyagi Cancer Center, Natori, Japan; 11 Department of Radiation Oncology, Iwate Prefectural Iwai Hospital, Ichinoseki, Japan; 12 Department of Radiation Oncology, Sendai Medical Center, Sendai, Japan; 13 Department of Radiation Oncology, Iwate Prefectural Ofunato Hospital, Ofunato, Japan

**Keywords:** Radium-223 dichloride, Bone metastasis, Castration-resistant prostate cancer, Radioisotope therapy

## Abstract

**Objective(s):**

In the treatment of castration-resistant prostate cancer (CRPC) with bone metastases, radium-223 dichloride (Ra-223) is the only bone-targeted drug that shows survival benefits. Completing six courses of Ra-223 treatment is thought to be associated with better patient survival, but this treatment has a relatively high rate of acute adverse events.

**Methods:**

This retrospective study included 85 patients from 12 institutions in Japan to investigate the clinical significance of the completion of Ra-223 treatment and acute adverse events in CRPC patients.

**Results:**

Six courses of Ra-223 treatment were completed in 65.9% of the patients. Grade 3 or higher acute adverse events were observed in 27.1% of patients. The prostate specific antigen and alkaline phosphatase declined at 26.9% and 87.9%, respectively. The overall survival rates at 12 and 24 months were 80.7% and 63.2%, respectively. Both completion of six courses of Ra-223 treatment and absence of grade 3 or higher acute adverse events were associated with longer overall survival. In univariate analysis, factors related to the history of treatment (five or more hormone therapy agents and cytotoxic chemotherapy) and hematological parameters (Prostate specific antigen (PSA) doubling time, alkaline phosphatase, hemoglobin, albumin, and serum calcium) were associated with completing six courses of Ra-223 treatment without experiencing grade 3 or higher acute adverse events. Multivariate analysis showed that a history of chemotherapy, PSA doubling time, hemoglobin, and serum calcium showed statistical significance. We built a predictive score by these four factors. Patients with lower scores showed higher rates of treatment success (p<0.001) and longer overall survival (p<0.001) with statistical significance.

**Conclusions:**

Accomplishing six courses of Ra-223 treatment without grade 3 or higher acute adverse events was a prognostic factor in patients with mCRPC treated with Ra-223. We built a predictive score of treatment success and need future external validation.

## Introduction

 In the treatment of prostate cancer, castration-resistant prostate cancer (CRPC) is defined as biochemical or radiographic disease progression, although castrate serum testosterone levels of <50 ng/dl or 1.7 nmol/l is achieved (1). Some patients have bone metastases at the time of diagnosis of CRPC, and 33% of CRPC patients who do not have bone metastases at the time will develop bone metastasis within two years (2). Radium-223 dichloride (Ra-223) is the only bone-targeted drug that shows survival benefits in metastatic CRPC (mCRPC) patients (3, 4). In a phase 3 trial with 921 patients (ALSYMPCA trial), patients treated with Ra-223 had a more prolonged overall survival (OS) than those treated with placebo (median OS: 14.9 months vs. 11.3 months, p<0.001) (5). 

 In Ra-223 treatment, completion of five or six courses of Ra-223 treatment is shown to be associated with a longer OS (6). Recently, some studies have reported predictive factors for completing Ra-223 treatment, which can be a surrogate marker for OS (7). In contrast, Ra-223 treatment causes various adverse events. In an international early access program, grade 3 and 4 treatment-related adverse events occurred in 11% and 1% of patients, respectively (8). A recent study by Turco et al. showed that patients who discontinued Ra-223 treatment due to toxicity had a shorter OS than those who discontinued Ra-223 treatment due to other causes (9). Therefore, it is assumed that patients will benefit from completing six courses of Ra-223 treatment without experiencing severe adverse events.

 In this study, we retrospectively analyzed the real-world data of patients treated with Ra-223 in multiple institutions in Japan. This study aimed to reveal the effect of completion of Ra-223 treatment and acute adverse events on patient outcomes and establish a predictive scoring system of treatment success.

## Methods

***Ethical approval and consent participate***

 All procedures performed in studies involving human participants were in accordance with the ethical standards of the institutional and/or national research committee and with the 1964 Helsinki declaration and its later amendments or comparable ethical standards. This study was approved by the Tohoku University Hospital Institutional Review Board (2021–1-585) as a central institutional review board of all of facilities. Informed consent for each patient was waived because this is a retrospective observatory study. Information on the study was presented on the institutional website, and the right to decline participating in this study was indicated for each patient.

***Patients***

 This study included 85 patients who received the first course of Ra-223 between January 2017 and January 2021 at 12 institutions in Japan. Data were collected from hospital records, including patient characteristics, treatments, imaging studies, hematological examinations, and follow-up information. Pre-treatment hematological evaluation was performed within seven days of starting Ra-223 treatment. PSA doubling time was calculated in an appropriate period within three months before the start of Ra-223 treatment, considering the clinical course including change of medication in the period. The data cutoff point was set on September 30, 2021.

***Treatment***

 All procedures performed in studies involving human participants were in accordance with the ethical standards of the institutional and/or national research committee and with the 1964 Helsinki declaration and its later amendments or comparable ethical standards. This study was approved by the Tohoku University Hospital Institutional Review Board (2021–1-585) as a central institutional review board of all of the facilities. Informed consent for each patient was waived because this is a retrospective observatory study. Information on the study was presented on the institutional website, and the right to decline participating in this study was indicated for each patient.

***Evaluation***

 Baseline status, treatment effects, and acute adverse events were evaluated through a retrospective review of patient records. The baseline extent of disease (EOD) was mainly assessed by bone scintigraphy. The status of Ra-223 treatment completion and the reasons for suspending Ra-223 treatment were recorded. Blood tests, including prostate specific antigen (PSA) and alkaline phosphatase (ALP) levels, were used to assess the treatment effect 12 weeks after the first Ra-223 treatment. Acute adverse events that correlated with Ra-223 treatment until 12 weeks after the last Ra-223 treatment were evaluated using the Common Terminology Criteria for Adverse Events version 5.0. In this study, treatment success was defined as six courses of Ra-223 treatment without grade 3 or higher acute adverse events. Predictive clinical, hematological, and radiological factors for treatment success were investigated using logistic regression analysis. Because of a small number of events, we used a least absolute shrinkage and selection operator (LASSO) model to determine parameters for constructing a predictive score for treatment success. In a LASSO analysis, parameters with a p-value smaller than 0.05 were analyzed, and missing blood test parameters were filled using the median values. Survival curves were generated using the Kaplan-Meier method, and the log-rank test was used to evaluate the different groups. In each analysis, a p-value smaller than 0.05 was considered significant. For statistical analyses, JMP® Pro 16.2.0 (SAS Institute Inc.) was used.

## Results

**Figure 1:**
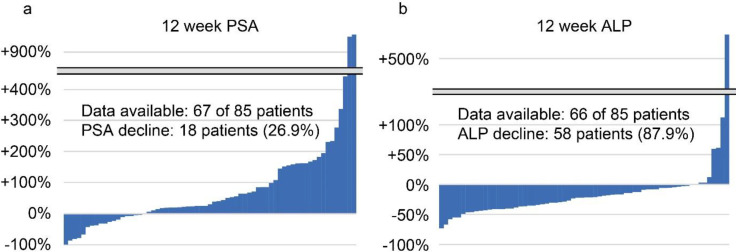
Waterfall plots of prostate specific antigen and alkaline phosphatase at 12 weeks from the start of Ra-223 treatment. PSA: prostate specific antigen, ALP: alkaline phosphatase

 A total of 85 patients from 12 institutions were included in this study. Patient characteristics are shown in Table 1. The median period from diagnosis of prostate cancer to the first Ra-223 treatment was 48.7 months (range: 9.0–218.5 months). Fifty-six patients (65.9%) completed six courses of Ra-223 treatment. The remaining 29 patients (34.1%) discontinued Ra-223 treatment for adverse events (16 patients, 18.8%), disease progression (11 patients, 12.9%), or unknown reasons (2 patients, 2.4%). Twelve weeks after the start of Ra-223 treatment, 81 of 85 patients were alive. PSA and ALP levels were evaluated at 12 weeks in 67 and 66 patients, respectively. Figure 1 shows waterfall plots for PSA and ALP.

**Table 1:** Patient characteristics. Values with an asterisk (*) are median values with ranges. ECOG: Eastern Cooperative Oncology Group, PSA: prostate specific antigen

	Number of patients (%)
**Age [years]**	74.7 (52–89.4)*
**Pathology of primary disease**	
Adenocarcinoma	72 (84.7%)
Ductal cell carcinoma	1 (1.2%)
Unknown	12 (14.1%)
Time from diagnosis to development of bone metastasis [months]	0.2 (0–168.3)*
Time from diagnosis to Ra-223 treatment [months]	48.7 (9–218.5)*
**History of treatment**	
Hormone therapy	85 (100.0%)
Cytotoxic chemotherapy	32 (37.6%)
Surgery	9 (10.6%)
Radiotherapy	21 (24.7%)
**ECOG performance status**	
0	38 (44.7%)
1	40 (47.1%)
2	5 (5.9%)
3	2 (2.4%)
**Extent of disease**	
No detectable in bone scintigraphy	6 (7.1%)
1	24 (28.2%)
2	24 (28.2%)
3	30 (35.3%)
4	1 (1.2%)
**Pain control**	
Without medication	51 (60.0%)
Non-opioid	15 (17.6%)
Weak opioid	10 (11.8%)
Strong opioid	9 (10.6%)
**Neurological symptom by bone disease**	
Yes	5 (5.9%)
No	80 (94.1%)
**Treatment facility**	
Medium volume (10–20 cases)	59 (69.4%)
Low volume (1–9 cases)	26 (30.6%)
**Concomitant treatment**	
Cytotoxic chemotherapy	0 (0.0%)
Hormonal therapy	60 (70.6%)
Bone modifying agent	58 (68.2%)
Steroid agent	31 (36.5%)
PSA [ng/ml]	18.9 (0.03–1388)*
PSA doubling time [days]	80.1 (16.6–∞)*
Alkaline phosphatase [U/l]	106 (22–659)*
Lactate dehydrogenase [U/l]	198 (17–820)*
Hemoglobin [g/dl]	12.7 (10.2–15.3)*
White blood cell [109/l]	6.5 (2.6–12.5)*
Neutrophil [109/l]	4.3 (1.8–9.3)*
Lymphocyte [109/l]	1.4 (0.4–4.1)*
Platelet [109/l]	201 (100–483)*
Creatinine [mg/dl]	0.79 (0.45–1.6)*
Albumin [g/dl]	4 (3.1–4.9)*
Calcium [mg/dl]	9.1 (6.9–10.1)*
C-reactive protein [mg/dl]	0.19 (0–4.74)*

**Figure 2:**
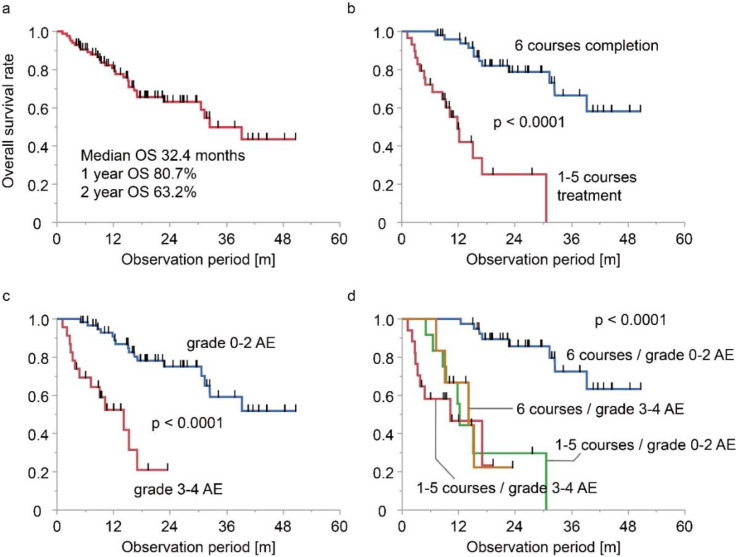
Survival curves plotted with Kaplan–Meier method of **a**: all patients and patients divided by **b**: treatment completion, **c**: occurrence of grade 3 or higher acute adverse events, and **d**: treatment completion and acute adverse events. Differences between groups were tested using log-rank test. OS: overall survival, AE: adverse event

 At the time of data cutoff, 56 of 85 patients (65.9%) were alive. The median follow-up period was 15.2 months for all patients and 19.1 months for live patients. The median survival period estimated by the Kaplan–Meier method was 32.4 months (Figure 2a). The OS rates at 12 and 24 months were 80.7% and 63.2%, respectively. Patients who completed six courses of Ra-223 treatment had a longer OS than those who did not complete the treatment course (Figure 2b; p<0.001, log-rank test).

 Acute adverse events of grade 2 or higher within three months of the last Ra-223 treatment were observed in 53 patients (62.4%). Grade 3 or higher adverse events were observed in 23 (27.1%) patients. Among these, 17 patients (20.0%) experienced hematological adverse events including anemia (12.9%), lymphocytopenia (9.4%), thrombocytopenia (5.9%), and neutropenia (1.2%), and 8 patients (9.4%) experienced non-hematological adverse events including fatigue (7.1%) and nausea (4.7%). Four patients (4.7%) experienced grade 4 adverse events, including a platelet decrease of less than 25,000/ml (3.5%) and anemia requiring transfusion (2.4%). Patients with grade 3 or higher adverse events showed a shorter OS than those with grade 2 or lower acute adverse events (Figure 2c; p<0.001, log-rank test). Figure 2d shows the Kaplan–Meier curves divided by treatment completion and grade 3 or higher acute adverse events. Fifty patients (58.8%) who completed six courses of Ra-223 treatment without grade 3 or higher acute adverse events had the longest OS, which was statistically significant.

 Additional analyses were conducted to investigate predictive factors for the completion of six courses of Ra-223 treatment without grade 3 acute adverse events. Table 2 shows the logistic regression analysis of pre-treatment parameters for predicting the completion of six courses of Ra-223 treatment without grade 3 or higher acute adverse events. In univariate analysis, factors related to the history of treatment (five or more hormone therapy agents and cytotoxic chemotherapy) and hematological parameters (PSA doubling time, ALP, hemoglobin, albumin, and calcium) were associated with treatment success. Of these parameters, LASSO analysis determined four significant parameters, including a history of cytotoxic chemotherapy, PSA doubling time, hemoglobin, and calcium (Table 3). One point was allotted to each parameter, where hemoglobin and calcium values were cut off at the lower limit of normal (13.0 g/dl for hemoglobin and 8.5 mg/dl for calcium), and the total points (0 to 4) were calculated (Table 4). 

**Table 2:** Logistic regression analysis for 6 courses treatment of Ra-223 without Grade 3 or higher acute adverse events (AE). For continuous variables, values are presented as median with range. Categorical variables were treated as binary values. ECOG: Eastern Cooperative Oncology Group, PSA: prostate specific antigen

	**6 courses of Ra-223** **without G3–4 AE**	**1–5 courses of Ra-223** **or G3–4 AE**	**Hazzard ratio (95% CI)**	**p-value**
**Age [years]**	74.1 (61.6–85.5)	75.7 (52.0–89.4)	0.99 (0.93-1.06)	0.84
**Pathology: adenocarcinoma**	82.00%	88.60%	0.59 (0.17-2.09)	0.41
**Time to development of bone metastasis: <3 months**	26.00%	54.30%	2.40 (0.96-6.00)	0.06
**History of treatment**				
Hormone therapy: ≥5 agents	40.00%	68.60%	0.31 (0.12-0.76)	0.01
Cytotoxic chemotherapy: yes	24.00%	57.10%	0.24 (0.09-0.60)	0.003
Surgery: yes	14.00%	5.70%	2.69 (0.52-13.79)	0.24
Radiotherapy: yes	18.00%	34.30%	0.42 (0.15-1.15)	0.09
Time from diagnosis to Ra-223 treatment [months]	47.4 (9.0–218.5)	52.2 (10.1–175.4)	1.00 (0.99-1.01)	0.67
Treatment facility: medium-volume center	68.00%	71.40%	0.85 (0.33-2.18)	0.74
ECOG performance status: 0	48.00%	40.00%	1.38 (0.58-3.32)	0.47
Extent of disease: 0–2	70.00%	54.30%	1.96 (0.80-4.83)	0.14
Pain control with medication	40.00%	40.00%	1.00 (0.41-2.42)	1.00
Presence of neurological symptom by bone disease	6.00%	5.70%	1.02 (0.16-6.46)	0.98
**Concomitant treatment**				
Cytotoxic chemotherapy: yes	0.00%	0.00%		
Hormonal therapy: yes	70.00%	71.40%	0.93 (0.36-2.41)	0.89
Bone modifying agent: yes	70.00%	65.70%	1.22 (0.48-3.07)	0.68
Steroid agent: yes	30.00%	45.70%	0.51 (0.21-1.25)	0.14
**Hematological parameters**				
PSA [ng/ml]	6.9 (0.0–176.1)	95.6 (2.6–405.9)	1.00 (0.99-1.00)	0.06
PSA doubling time <60 days	23.30%	52.90%	0.27 (0.10-0.72)	0.01
Alkaline phosphatase [U/l]	101.5 (22.4–368.6)	108.2 (46.6–654.9)	1.00 (0.99-1.00)	0.02
Lactate dehydrogenase [U/l]	194.0 (17.0–439.0)	210.0 (106.0–820.0)	1.00 (0.99-1.00)	0.09
Hemoglobin [g/dl]	13.2 (10.3–15.3)	11.9 (10.2–15.1)	2.11 (1.37-3.25)	0.001
White blood cell [109/l]	6.4 (3.7–12.5)	6.7 (2.6–12.4)	1.00 (1.00-1.00)	0.66
Neutrophil [109/l]	4.2 (2.0–7.7)	5.1 (1.8–9.3)	1.00 (1.00-1.00)	0.13
Lymphocyte [109/l]	1.6 (0.5–4.1)	1.2 (0.4–3.2)	1.00 (1.00-1.00)	0.13
Platelet [109/l]	210 (134–307)	197 (100–483)	1.00 (1.00-1.00)	0.74
Creatinine [mg/dl]	0.83 (0.49–1.60)	0.78 (0.45–1.43)	4.37 (0.53-36.23)	0.17
Albumin [g/dl]	4.1 (3.3–4.9)	3.9 (3.1–4.5)	10.94 (2.35-51.03)	0.002
Calcium [mg/dl]	9.2 (8.5–10.1)	8.9 (6.9–9.7)	8.12 (2.25-29.32)	0.001
C-reactive protein [mg/dl]	0.19 (0.00–2.40)	0.18 (0.02–4.74)	0.66 (0.33-1.33)	0.25

**Table 3:** Multivariate analysis using a least absolute shrinkage and selection operator model

.	HR (95% CI)	p-value
≥5 agents of hormone therapy	0.66 (0.17-2.51)	0.54
History of cytotoxic chemotherapy	0.22 (0.06-0.83)	0.03
PSA doubling time <60 days	0.14 (0.04-0.54)	0.004
Alkaline phosphatase [U/l]	1.00 (0.99-1.00)	0.34
Hemoglobin [g/dl]	1.97 (1.11-3.50)	0.02
Albumin [g/dl]	1.09 (0.13-9.33)	0.94
Calcium [mg/dl]	6.74 (1.43-31.88)	0.02

**Table 4:** A predictive score of 6 courses treatment of Ra-223 without Grade 3 or higher acute adverse events

	**point**
History of cytotoxic chemotherapy	1
PSA doubling time <60 days	1
Hemoglobin < 13.0 g/dl	1
Calcium <8.5 mg/dl	1
**Total score**	4

**Figure 3:**
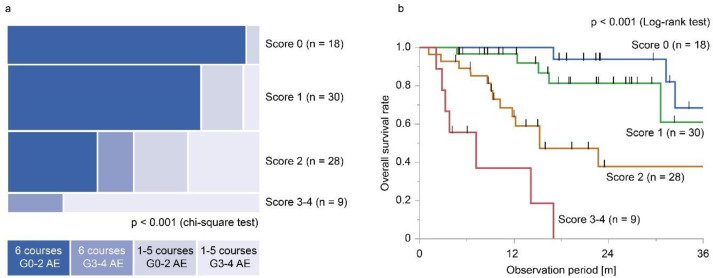
Predictive scores for Ra-223 treatment completion and acute adverse events (AE). **a**: Treatment success rate for each group and **b**: survival analysis for each group

 Patients were divided into four risk groups by the total points. Figure 3 shows the treatment success rates and survival curves for the four groups. The treatment success rates were 94.4%, 76.7%, 35.7%, and 0.0% for patients with 0, 1, 2, and 3-4 points, respectively (Figure 3a, p<0.001 with chi-square test). The area under the curve value for predicting treatment success was 0.839. The log-rank test showed a statistically significant difference in the survival curves of the four groups (Figure 3b, p<0.001). One-year survival rate of four groups were 100%, 96.7%, 63.9%, and 37.0%, respectively.

 To explore the background of hypocalcemia in the study population, additional analyses were performed on the association between hypocalcemia and the use of bone-modifying drugs. In patients with and without hypo-calcemia (serum calcium concentration below 8.5 mg/dl), the rate of bone-modifying drug use was 67.1% and 83.3%, respectively (p=0.38 by chi-square test). The mean calcium concentration at baseline was 9.0 mg/dl in bone-modifying drug users and 9.2 mg/dl in non-users, with no statistical significance (p=0.08 by t-test).

## Discussion

 In the current study, we demonstrated that both completion of six courses of Ra-223 treatment and the occurrence of grade 3 or higher acute adverse events were associated with OS in patients with mCRPC treated with Ra-223. We also built a predictive score for treatment completion without severe adverse effects and showed that the score discriminates treatment success well. In this study, we registered 85 patients from 12 institutes, and the median number of patients from a single institute was 4. In the univariate analysis, treatment experience (≥10 patients vs. <10 patients per institute) was not associated with treatment success. Considering that most of the past publications on Ra-223 treatment were from high-volume centers, information in this report might be helpful not only for high-volume centers but also for non-high-volume centers.

 The median OS of the study population was 32.4 months. In Japan, unlike most European and North American countries, Ra-223 is approved for both symptomatic and asymptomatic patients with mCRPC. In the current study, 60.0% of the patients did not have bone pain that required medication. This might be associated with a favorable OS compared to existing real-world outcomes (10,11), as an international early access program of Ra-223 showed that asymptomatic patients had a higher rate of treatment completion and a longer OS (12). In the treatment evaluation at 12 weeks from the start of Ra-223 treatment, the decline rates of PSA and ALP were 26.9% and 87.9%, respectively, which is consistent with existing reports (13, 14). 

 In Ra-223 treatment for bone metastases of mCRPC, many studies have shown that the completion of six courses of treatment is associated with a better OS (6, 10), which are consistent with our data. In terms of safety, 27.1% of the patients had grade 3 or higher acute adverse events. This is a relatively lower rate than in a phase 3 study of 226 Asian patients, where grade 3 or higher adverse events were reported in 46% of the patients (11). In an early access program of Ra-223 with 708 patients, including 19% of asymptomatic patients, grade 3 or 4 treatment-emergent adverse events were observed in 29% and 40% of the asymptomatic and symptomatic patients, respectively (12). Administration of Ra-223 at an early stage without bone pain may have contributed to the lower rate of adverse events. 

 Turco et al. recently reported that Ra-223 treatment discontinuation due to adverse events is related to worse outcomes than discontinuation due to disease progression (9). 

 In our study, patients with grade 3 or higher acute adverse events showed worse survival even after completing six courses of Ra-223 treatment, compared to those who completed six courses of Ra-223 treatment without grade 3 acute adverse events (median survival period: 14.1 months vs. not reached; p < 0.0001, log-rank test). These results suggest that both the completion of six courses of Ra-223 treatment and the absence of grade 3 or higher acute adverse events are important for patient prognosis.

 Based on these facts, we analyzed predictive factors of the completion of six courses of Ra-223 treatment without grade 3 or higher acute adverse events. Because the number of events was small, we employed LASSO as multivariate analysis and built a predictive score. We found that the history of cytotoxic chemotherapy, PSA doubling time, hemoglobin level, and calcium level could be predictors of the completion of six courses of Ra-223 treatment without grade 3 or higher acute adverse events. Among the four parameters, PSA doubling time reflects disease progression speed and has been reported as a predictor for the completion of Ra-223 treatment (15). A shorter PSA doubling time can cause disease progression in sites other than bone during the treatment period of Ra-223. In previous studies, the history of cytotoxic chemotherapy and hemoglobin concentration also have been reported to be predictors for the completion of five or six courses of Ra-223 treatment (7, 16–18). Considering that both represent accumulated treatment burden and bone marrow reserve, it may be favorable to introduce Ra-223 early in the course of treatment. However, there is a possibility that hematological toxicity caused by Ra-223 limits following treatment, including taxane agents. Further study for optimizing treatment sequencing is needed to solve this problem. We also found that higher serum calcium levels were associated with successful treatment. In patients with advanced cancer, hypercalcemia is caused by osteolytic bone metastases and parathormone-related proteins produced by tumor cells and is related to a poor prognosis. In contrast, in the treatment of prostate cancer, hypocalcemia is associated with bone-modifying drugs such as bisphosphonate and denosumab (19, 20). In our data, more patients treated with bone-modifying drugs showed hypocalcemia but there was no statistical difference. Further research would be needed on this issue. Based on the result of the multivariate analysis, we constructed a predictive score and showed that patients with lower scores are more likely to not only complete six courses of treatment without experiencing severe adverse events, but also have a longer OS period. Although this exploratory analysis requires external validation, it might help in daily clinical decision-making.

 This study has some limitations. First, there were some data defects, especially in the treatment history, because this was a multicenter retrospective study. Second, the dataset size was not large enough to conduct multivariate analysis to determine the independent predictors of treatment success. Our analysis of the predictive score was exploratory and needs to be evaluated using an independent dataset. Third, 60.0% of patients did not use any analgesic agents, which might make it difficult to interpret the results of the current study in clinical settings outside Japan.

## Conclusion

 Accomplishing six courses of Ra-223 treatment without grade 3 or higher acute adverse events was a prognostic factor in patients with mCRPC treated with Ra-223. We built a predictive score of treatment success and need future validation. This score needs to be confirmed prospectively in an independent cohort.
